# Progress

**Published:** 1887-12

**Authors:** Ella Wheeler Wilcox


### PROGRESS.

BY ELLA WHEELER WILCOX.

Let there be many windows in your soul,
  That all the glory of the universe
May beautify it. Not the narrow pane
  Of one poor creed can catch the radiant rays
That shine from countless sources. Tear away
  The blinds of superstition; let the light
Pour through fair windows broad as truth itself
  And high as God.

Why should the spirit peer
  Through some priest curtained orifice, and grope
Along dim corridors of doubt, when all
  The splendor from unfathomed seas of space
Might bathe it with golden waves of love?
  Sweep up the debris of decaying faiths;

Sweep down the cobwebs of worn-out beliefs,
And throw your soul wide open to the light
Of Reason and of Knowledge. Tune your ear
To all the worldless music of the stars
And to the voice of nature, and your heart
Shall turn to truth and goodness, as the plant
Turns to the sun. A thousand unseen hands
Reach down to help you to their peace-crowned heights,
And all the forces of the firmament
Shall fortify your strength. Be not afraid
To thrust aside half truths and grasp the whole.

